# Computational and biological characterization of fusion proteins of two insecticidal proteins for control of insect pests

**DOI:** 10.1038/s41598-018-23138-3

**Published:** 2018-03-19

**Authors:** Shaista Javaid, Sehrish Naz, Imran Amin, Georg Jander, Zaheer Ul-Haq, Shahid Mansoor

**Affiliations:** 1 Agricultural Biotechnology Division, National Institute for Biotechnology and Genetic Engineering (NIBGE), P. O. Box 577, Jhang Road, Faisalabad, Pakistan; 2 Pakistan Institute of Engineering and Applied Sciences (PIEAS), Nilore, Islamabad, Pakistan; 3 Institute of Molecular Biology & Biotechnology, University of Lahore, 1-KM Defence Road, Lahore, Pakistan; 4 Dr. Panjwani Center for Molecular Medicine and Drug Research, International Center for Chemical and Biological Sciences, University of Karachi-, 75270 Karachi, Pakistan; 5 Boyce Thompson Institute, 533 Tower Road, Ithaca, NY 14853 USA; 6 Department of Drug Design and Pharmacology, University of Copenhagen, Copenhagen, Denmark

## Abstract

Sucking pests pose a serious agricultural challenge, as available transgenic technologies such as *Bacillus thuringiensis* crystal toxins (Bt) are not effective against them. One approach is to produce fusion protein toxins for the control of these pests. Two protein toxins, Hvt (ω-atracotoxin from *Hadronyche versuta*) and onion leaf lectin, were translationally fused to evaluate the negative effects of fusion proteins on *Phenacoccus solenopsis* (mealybug), a phloem-feeding insect pest. Hvt was cloned both N-terminally (HL) and then C-terminally (LH) in the fusion protein constructs, which were expressed transiently in *Nicotiana tabacum* using a *Potato Virus X* (PVX) vector. The HL fusion protein was found to be more effective against *P. solenopsis*, with an 83% mortality rate, as compared to the LH protein, which caused 65% mortality. Hvt and lectin alone caused 42% and 45%, respectively, under the same conditions. Computational studies of both fusion proteins showed that the HL protein is more stable than the LH protein. Together, these results demonstrate that translational fusion of two insecticidal proteins improved the insecticidal activity relative to each protein individually and could be expressed in transgenic plants for effective control of sucking pests.

## Introduction

Crop yield can be increased to many fold by using chemical pesticides for the control of insect pests. However, the excessive use of chemicals causes destruction of non-target insects and development of resistance, along with environmental contamination. Since 1960, bio-insecticides have received considerable attention, due to being environment friendly and highly desirable alternatives for the control of insect-pathogenic microbes, viruses, and eelworms^1,2^.

Expression of two different proteins as a single translationally fused protein under the control of a single promoter has also gained much attention in the recent years^3–6^. This typically allows the one-step expression of transgenic lines, unlike the gene stacking with recombinant traits, multiple T-DNA and promoter insertions, and thereby coordinated expression of different recombinant proteins in plants. A number of fusion proteins have been designed and tested for the control of pests and pathogens. Usually, fusion constructs comprise δ-endotoxins from *B. thuringiensis* (Bt) and/or other plant defense-related proteins^7–9^. Bt is the most widely used bio-insecticide for the control of insect pests. These toxins specifically destroy the intestinal lining of target insects to kill them^10^. More than 35 million hectares are being cultivated with Bt crops globally in at least 13 different countries. There are many crops have been transformed genetically with Bt genes, including cotton, maize, eggplant, tobacco, potato, rice, and tomato. However, on a commercial scale, only cotton and maize have been transformed to have insecticidal activity^11^. Bt toxins are quite effective for the control of insects belonging to the family Lepidoptera and Coleoptera^12^. Bt toxins are considered to be most effective for controlling stem borers and root worms^13^. They are quite specific in their mode of action and are ineffective against many sucking pests including African rice green hoppers, rice thrips, white backed bugs, and green leaf hoppers^14,15^. Recently, targeted mutagenesis of the Cry51 protein and a fusion complex of Cyt2Aa with aphid gut binding peptide were found to make Bt toxins effective against *Lygus* spp.^16^ and aphids^17^, respectively. Nevertheless, there is a need to discover and study new proteins with insecticidal activity that is specific for the control of sucking pests, in particular phloem feeders.

Spider venom is a complex of many toxins that causes numerous neurological and biochemical changes in animals including mammals^18^. It contains a variety of toxic polypeptides that paralyze the prey prior to feeding. These polypeptide neurotoxins have ligand binding activity for neuronal ion channels and, to a lesser extent, neuronal receptors and presynaptic membrane proteins^19,20^. ω -Atracotoxin from *Hydronyche versuta* (Blue Mountains funnel web spider) is a calcium channel antagonist. It binds to insect calcium channels and blocks nerve impulses, ultimately leading to paralysis and death of the insect^21,22^. Multiple compounds from different spider venom have already been proved effective against insect pests belonging to order Lepidoptera and Hemiptera^23–25^.

Lectins belong to a superfamily of proteins that recognize and bind the carbohydrate moieties without altering their covalent structure. Some plants, in particular legumes and cereals have high concentrations of lectins because of their interaction with storage proteins^26,27^. As plant defense proteins, lectins play a biological role in the recognition of different proteins, and exhibit their insecticidal, fungicidal and anti-microbial properties^28^. At the laboratory scale, lectins are used to study different cellular mechanisms, including apoptosis, proliferation, cell arrest, cell metastasis, and recognition of different glycans in microarray^29,30^. *Galanthus nivalis* agglutinin (GNA, or snowdrop lectin) is a plant lectin that shows its limited insecticidal activity for lepidopteran larvae. It has been shown in artificial diet and transgenic plant studies that GNA reduces larval weight gain and slows down the developmental rate of first instar *Lacanobia oleracea* larvae^31,32^. This is achieved by binding of the lectin to the gut epithelium surface glycoproteins^33^. However, the exact mechanism by which GNA effects insect pests is still unclear. The ability of GNA to cross the gut epithelium makes this protein a potential carrier to deliver fused peptides to the circulatory system of target insect species.

Transient expression studies were carried out using the potato virus X (PVX) expression system. This is a quick and reliable system for studying different genes *in planta*^34–37^. To serve as expression vector, the PVX genome was cloned behind a T7 RNA polymerase promoter or a 35 S promoter derived from *Cauliflower mosaic virus* (CaMV)^17^.

We have shown previously that the expression of Hvt and lectin in transgenic plants from phloem-specific promoters protects these plants against multiple sucking pests^25^. Here we have explored the significance of translational fusion of Hvt and lectin that was transiently in tobacco using the PVX expression vector. Both computational tools and insect bioassays using *Phenacoccus solenopsis* (mealybug) as a model were used to assess the stability and insecticidal potential of fusion proteins.

## Results

**Figure 1:**
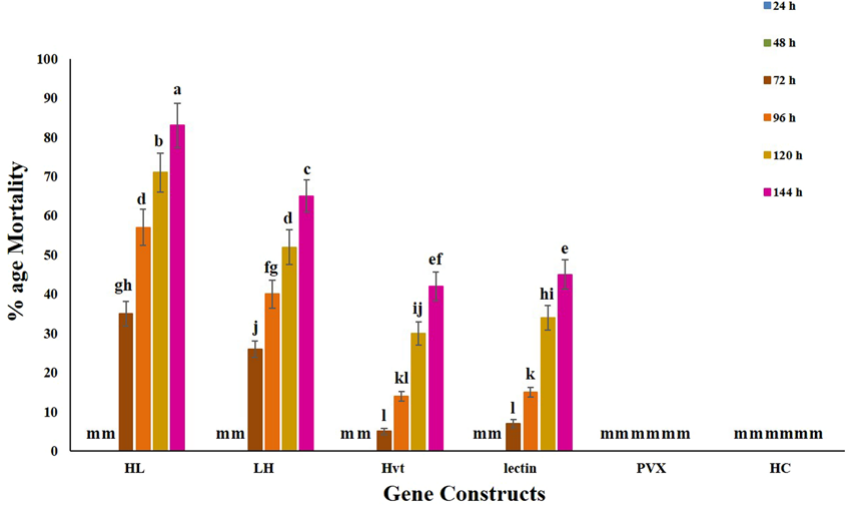
Graph showing mean percent mortality of *P. solenopsis* due to the effects of Hvt, lectin and translationally fused proteins (HL and LH) over time. No insect mortality was observed on PVX empty vector and healthy control (HC) plants. Different letters above the bars indicate *p* < 0.05, ANOVA followed by Tukey’s HSD test. Each bar represents the mean value of the mortality data collected from five plants during three replicates of the bioassay experiment. The error bars show the standard deviation of the mean.

**Figure 2:**
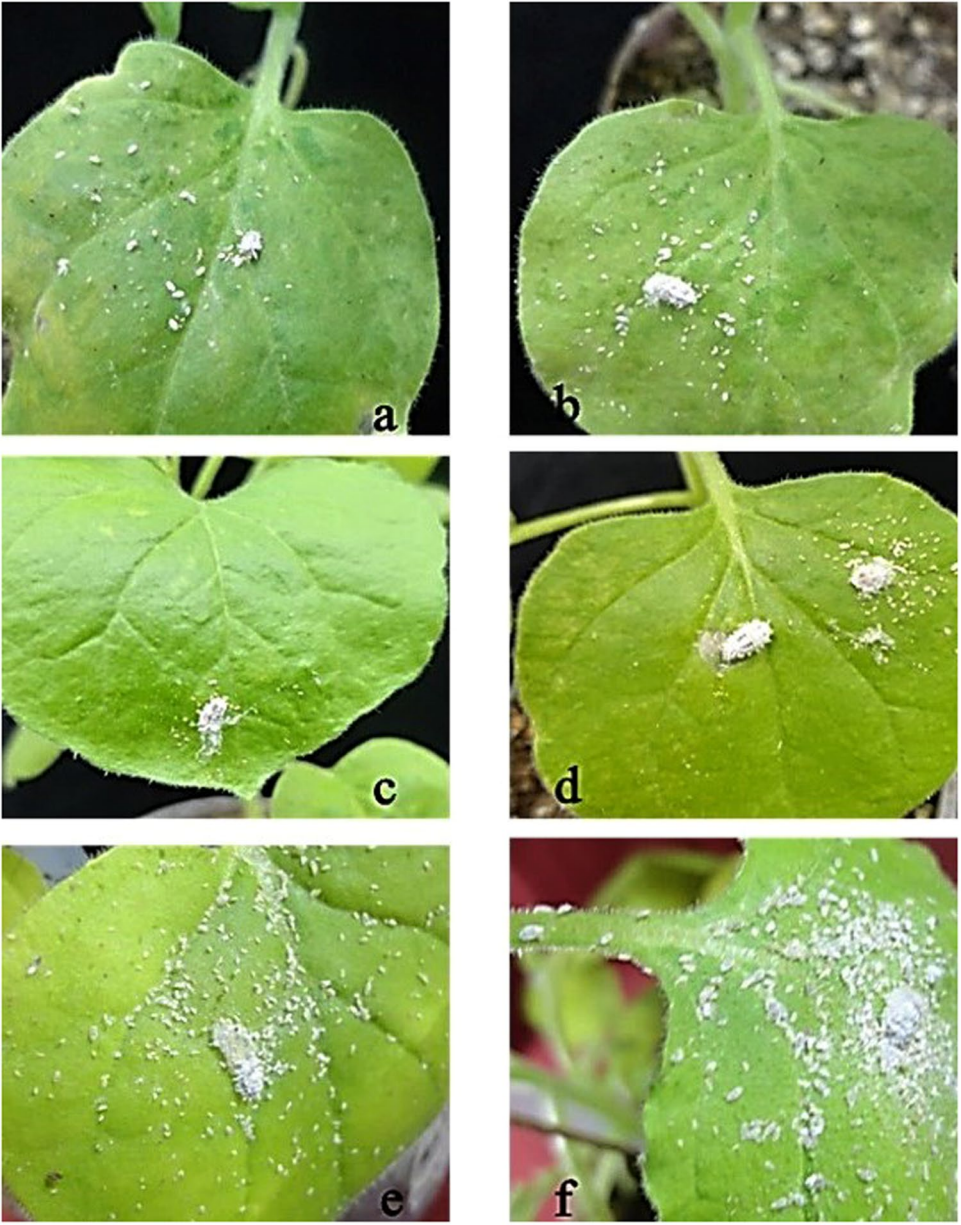
Representative pictures of bioassays performed with *P. solenopsis* feeding on transiently expressed toxin proteins and control tobacco plants (**a**) plants expressing Hvt protein (**b**) plants expressing lectin protein (**c**) plants expressing HL (**d**) plants expressing LH (**e**) healthy control (**f**) PVX control.

*Nicotiana tabacum* (tobacco) plants infiltrated with PVX carrying protein constructs, along with the control plants, were kept in cages and exposed to mealybug feeding. Initially, mealybugs took time to acclimatize with the new environment, and during the first 48 h we did not observe any lethal effects. After two days, mealybugs appeared lethargic, with very slow movements. Nymphs in particular showed early effects of the toxins. They started to lose body mass and had jerky and shaky movements. After three days, 35% of nymphs were found dead on the plants expressing HL (Hvt-lectin) toxin, and 26% on LH (lectin-Hvt) plants. In contrast, plants expressing the parent toxins, Hvt and lectin, caused only 5 and 7% mortality, respectively. Higher mortality was recorded after 6 days for all transgenes: HL (83%), LH (65%), Hvt (42%), and lectin (45%) (Figs 1 and 2).

As some insects were found to escape from the plants, we could not get 100% mortality data. Some of the adult individuals multiplied on the plants, but the nymphs could not grow or move to the next instar due to the effect of the toxic proteins. The ovisac of the multiplying adult mealybugs was found to be normal. The dead insects showed the symptoms of brown discoloration of their bodies along with reduction in body mass.

In contrast, on healthy control and PVX control plants, the mealybugs grew and multiplied normally. Young nymphs developed into adults and adult individuals multiplied with true ovisac. All the phenotypes were normal, including gain of body mass and multiplication.

### Computational studies

Computational studies were used to investigate the conformational changes in the fusion proteins and to validate the experimental results that the fusion protein expressing Hvt at the N-terminus (HL) is more stable and lethal to the insects compared to the protein expressing Hvt at the C-terminus (LH). Moreover, significant factors affecting the biological properties of the fusion proteins were also predicted by these *in-silico* studies.

### Structure prediction of proteins

**Figure 3:**
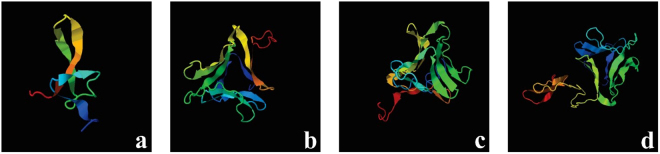
3D tertiary structures of native and fusion proteins predicted by I-TASSER (**a**) Hvt (**b**) lectin (**c**) HL (**d**) LH.

The change in orientation of Hvt and lectin in the fusion constructs caused large differences in the tertiary structure of both parent proteins, Hvt and lectin (Fig. 3). Therefore, *in silico* tools were utilized to predict the most suitable 3D model for both parent and fusion proteins. Initially, a 20 ns simulation was performed for each of the above-mentioned proteins to generate a refined structure, which was followed by additional analysis to predict the core factors responsible for the dynamics and stability of one construct relative to the other.

### Ligand binding site of protein

In this study, we dealt with the proteins having ligand binding properties. The ligand binding sites of the parent and fusion proteins were predicted by I-TASSER (COACH) tool. COACH is a meta-server approach that combines multiple function annotation results from the COFACTOR, TM-SITE, and S-SITE programs^38–41^.

**Figure 4:**
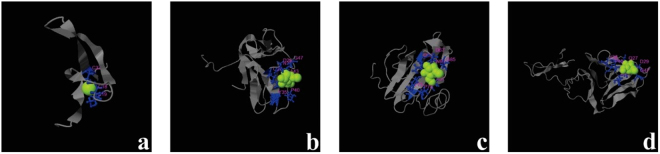
3D structures of ligand binding sites of native and fusion proteins predicted by I-TASSER (COACH) (**a**) Hvt (**b**) lectin (**c**) HL (**d**) LH.

In case of Hvt, I-TASSER-COACH server predicted P7, C23 and Q21 as the crucial residues involved in the ligand binding site, with a cluster size of 138 Å. For lectin, the Q28, D30, N32 Y36, P41, A44, and G48 residues were found to be a crucial part of the ligand binding site, with a cluster size of 34 Å. In the case of fusion proteins, the ligand binding site of HL consists of, Q105, D107, N109, Y113,, A119, A123, and N124 with a cluster size of 37 Å, while for LH protein, it consists of Q59, D61, N63, Y67, A73, A76, and R80 with a cluster size of 24 Å (Fig. 4). This also shows that the ligand binding site of fusion protein has conformational differences compared to the parent proteins. The cluster size for fusion proteins is reduced compared to the parent proteins. In a comparison of the two fusion proteins, HL has a larger cluster size than LH.

### Evaluation and refinement of modelled structures

**Figure 5:**
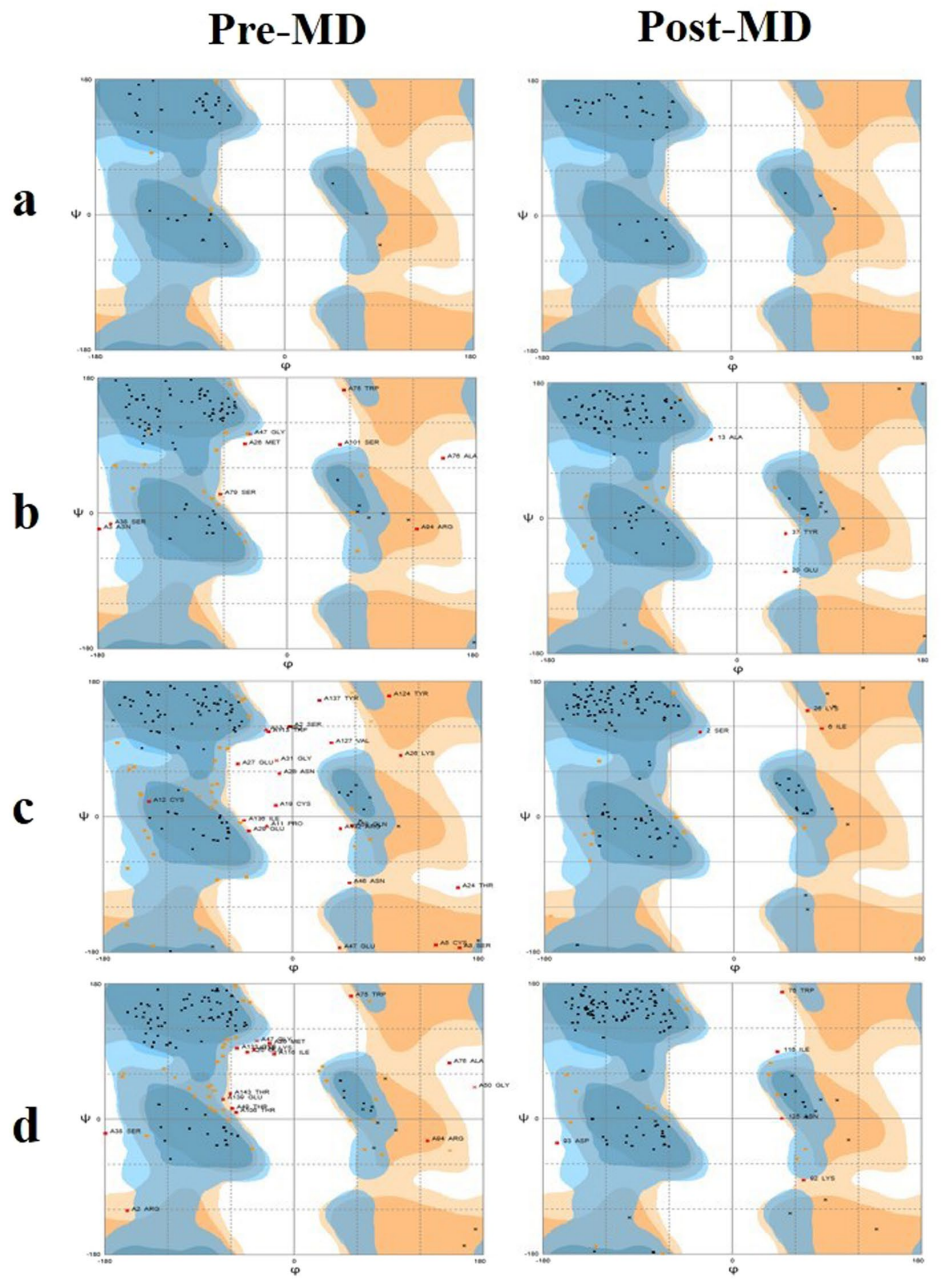
Comparative Ramachandran plots of all four proteins before and after MD simulation (**a**) Hvt (**b**) lectin (**c**) HL (**d**) LH. The dark blue color presented the general favored region, pale blue shows allowed region while dark and pale orange displays glycine favored and allowed regions respectively. Outlier region is depicted with white color.

The accuracy of modelled structures was evaluated by calculating Ramachandran plots for each of the four proteins, before and after simulation. A comparative analysis is summarized and plots are displayed (Table 1, Fig. 5). The data showed the overall refinement of modelled structures as the number of amino acids in favored and allowed regions were improved, while residues in outlier region were reduced after a 20 ns simulation. This showed the validation and accuracy of post-molecular dynamics (MD) modelled structures over pre-MD modelled structures. Considering all above model evaluations, it was inferred that the post-MD structures were of good quality and suitable for further analysis.

**Table 1:** Comparative Ramachandran plot analysis of pre-and post MD modelled structures with percentage of residues in favored, allowed and outlier regions.

Proteins		Favored region (%)	Allowed region (%)	Outlier region (%)
Hvt	Pre-MD	89.7	10.3	0.0
	Post-MD	100	0.0	0.0
Lectin	Pre-MD	75.0	16.7	8.3
	Post-MD	86.1	11.1	2.8
HL	Pre-MD	61.0	24.0	15.1
	Post-MD	89.7	8.2	2.1
LH	Pre-MD	64.4	24.7	11.0
	Post-MD	86.3	10.3	3.4

### Root mean square deviation

**Figure 6:**
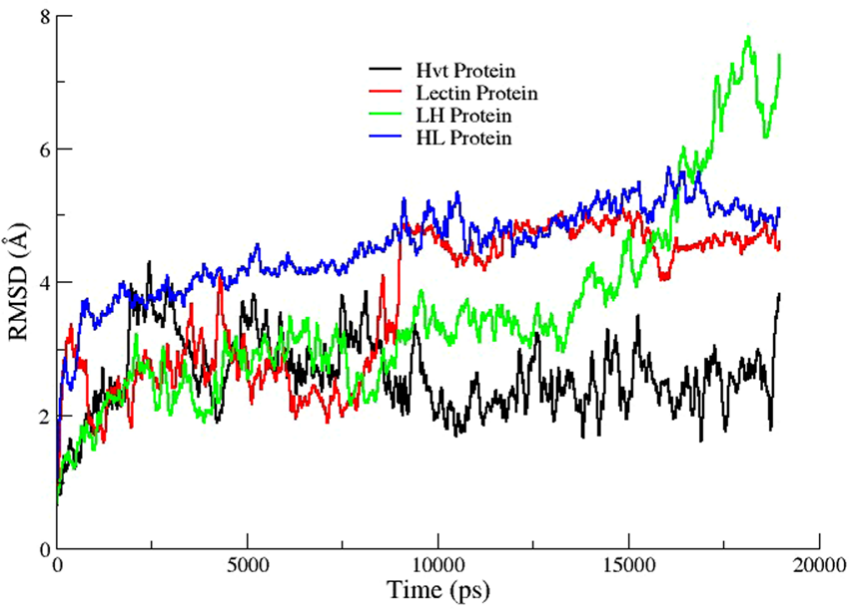
RMSD of backbone C-α atoms of both parent proteins in black (Hvt) and red (Lectin) color along with their two fusion constructs in green (LH) and blue (HL) color as a function of time (ns).

Initially, differences in the positions of backbone atoms were calculated to investigate the stability and deviation of each protein from its initial structure. The root mean square deviation (RMSD) plots (Fig. 6) revealed that HL fusion construct (Avg RMSD 4.0 ± 0.5 Å) exhibits a steady-state structure with very low deviations, while the LH fusion construct shows a drastic increase in RMSD starting from 0.5 to 7.0 Å. Similarly, both parent proteins Hvt and lectin displayed a strong deviation during initial 10 ns, while both proteins were stabilized during last 10 ns around 2.0 ± 0.3 and 4.3 ± 0.2 Å, respectively.

### Radius of gyration (Rg)

**Figure 7:**
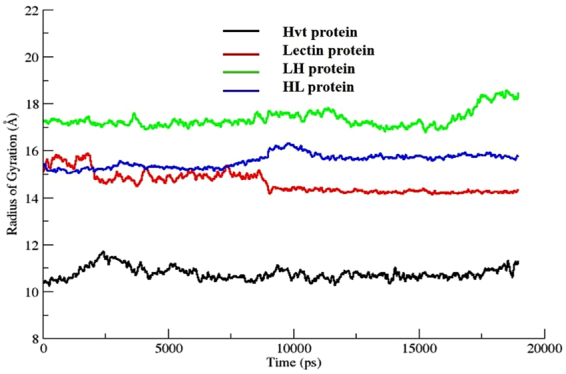
The radius of gyration plots for all four proteins with respect to time (ps) presenting the comparative compactness.

With the aim of evaluating a stable orientation of the parent protein from the two constructs, the compactness of all four proteins was studied. In this regard, the size of the protein was calculated via a radius of gyration plot, which determined the mean positional distances of atoms from the center of mass. Figure 7 illustrates the significant changes in the compactness level of all the four proteins in terms of their Rg values. Higher Rg values demonstrate less compactness (more unfolded) with more conformational entropy in the structure while low Rg values show more compactness and stability in the structure (more folded). The average values of Rg plots showed more compactness in the Hvt parent protein at 10.5 ± 0.2 Å than in the lectin parent protein, which showed larger Rg values around 14.0 ± 0.2 Å. Similarly, the lower Rg values for HL construct (16.0 ± 0.2 Å) indicates greater overall compactness and structural stability of this construct compared to the LH construct (17.2 ± 0.2 Å).

### Hydrogen bond analysis

**Figure 8:**
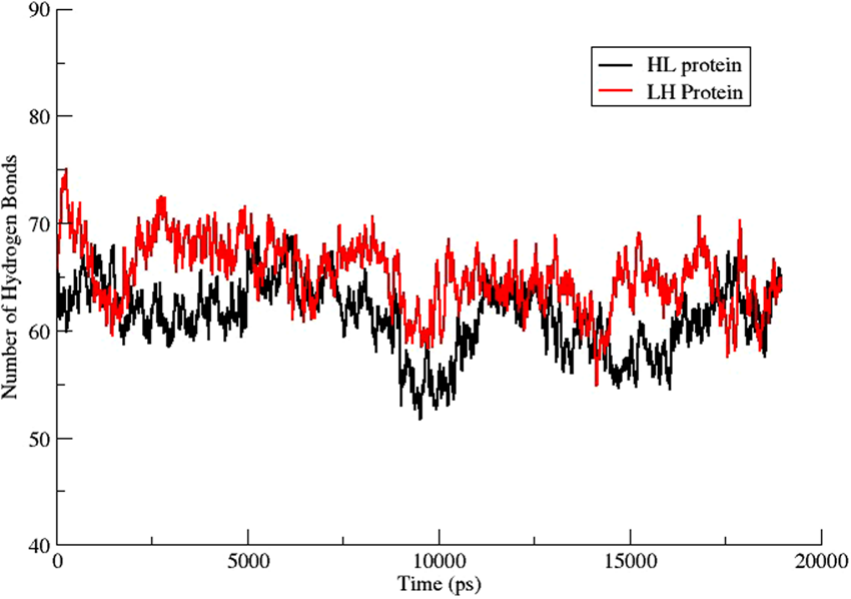
Comparative plot of total number of hydrogen bonds formed and deformed during entire 20 ns simulation among the residues of LH and HL fusion constructs.

For better understanding the underlying basis for the greater stability of the HL construct relative to the LH construct, the total number of hydrogen bonds formed and deformed during the entire simulation period was calculated. HL and LH showed a similar number of hydrogen bonds throughout our simulation, as plotted in Fig. 8. The average number of hydrogen bonds observed for the LH construct was 65, which dropped to 61 in the HL construct. Next, we counted the hydrogen bonds that presented more than 50% occupancy during the entire simulation. For HL, there were 32 such hydrogen bonds, and this number increased to 38 in the LH protein. From these results, we deduced that some factors other than overall hydrogen bonding are influencing the stability of these fusion constructs.

### Root mean square fluctuation

**Figure 9:**
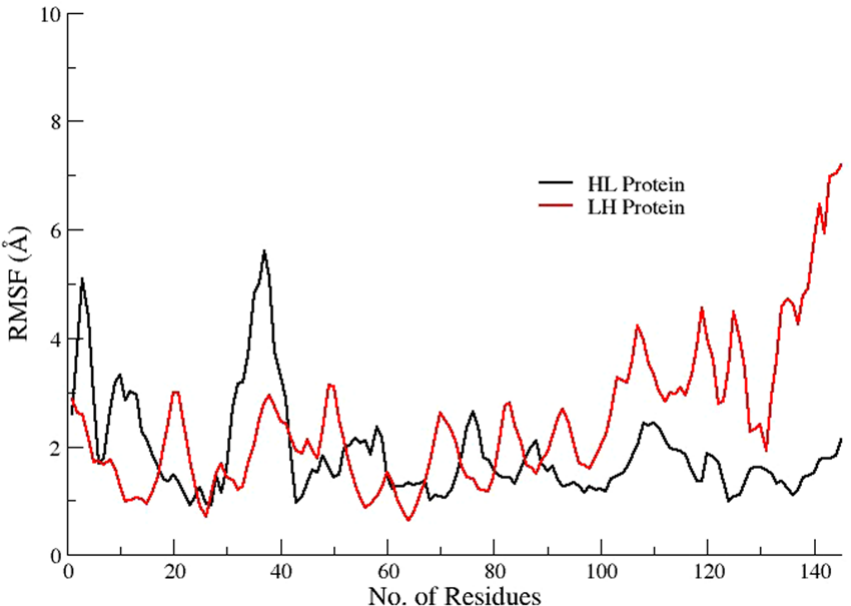
RMSF plots of both HL and LH fusion construct displaying the comparative stability of HL construct over LH by presenting less fluctuations in its residues.

To analyze the mean atomistic motions of separate residues, root mean square fluctuation (RMSF) of Cα atoms in both LH and HL fusion constructs were calculated using final trajectories. High RMSF values denote more flexibility (more conformational fluctuation), while low values show less fluctuations in the structure. As shown in Fig. 9, the fluctuations in the LH construct (2.5 ± 0.5) are larger, predicting more conformational changes. Comparatively, the RMSF plot of the HL construct has 1.8 ± 0.2 Å movement, demonstrating overall stability of this construct, with the exception of a few residues in N-terminal domain that show greater fluctuations. The results suggested that the orientation of the parent proteins in the HL construct are quite stable, with less structural changes than the other orientation in the LH construct.

### Secondary structure analysis

**Figure 10:**
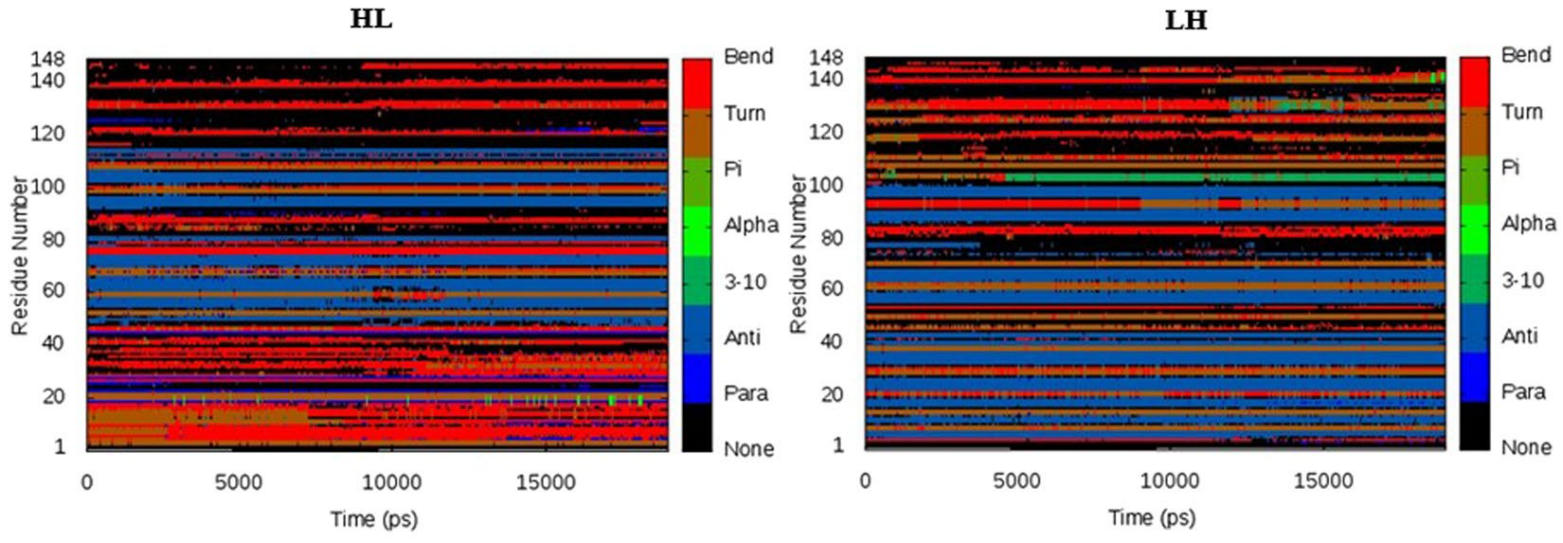
Comparative secondary structure plots of HL and LH construct presenting the drastic change in their secondary structure as a result of different orientation of parent proteins.

For the estimation of structural stability and fluctuations of residues in the formation of secondary structures, average secondary structure plots for both the HL and the LH fusion construct systems were calculated. The average secondary structure content and its position is presented in Fig. 10. Detailed investigation of both plots revealed drastic conformational differences between the HL and LH fusion proteins. The stretch of 40 residues present in the N and C-terminus of the HL construct were found to be engaged in the formation of bends and turns whereas the residues were changed to an anti-parallel β-sheet in the LH construct. It appears clearly from the plots that the major part of the secondary structure found in the LH protein is an anti-parallel β-sheet, while the HL construct has bends, turns and an anti-parallel β-sheet in its structure which might contribute to the stability of the HL construct.

### Structural dynamics study

**Figure 11:**
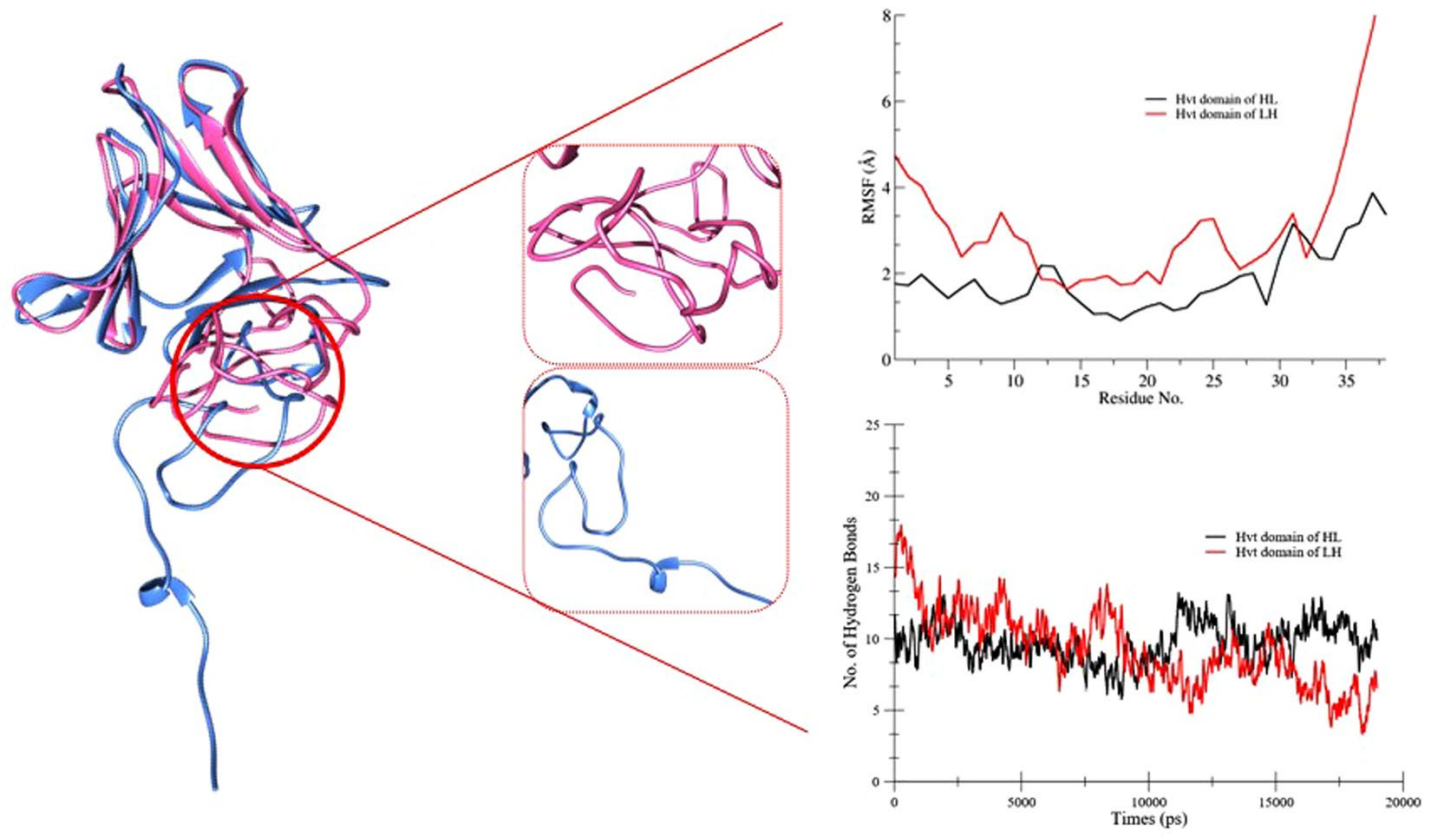
Left side presenting the superimposed 3D ribbon diagram of both HL (pink) and LH (pale blue) construct highlighting the structural transition of Hvt domain while right side displaying the comparative RMSF and hydrogen bond plots of Hvt domain of both constructs.

The ambiguous picture developed by analyzing hydrogen bond and secondary structure plots suggested further visualization of trajectories to explore the key players responsible for the structural differences between the HL and LH constructs. The molecular visualization of the trajectories brought forth an interesting finding, predicting a crucial role of the Hvt domain in the stability of the whole protein. Superimposition of post-MD structures of the HL and LH constructs showed that the structure of the lectin domain remains rigid and stable through the entire simulation, while the Hvt domain continues changing. The Hvt domains of both constructs were observed in different conformations having different secondary structures. The Hvt part of the HL protein contains both bends and turns, while the LH has just bends with very few turns and helices in its Hvt domain. We then calculated RMSF and hydrogen bonds found in this region of both constructs to predict whether the Hvt domain has any role in the stability of the fusion proteins (Fig. 11). Our analysis showed that the Hvt region of the HL protein is stable through the entire simulation, with low RMSF values around 2 Å because of the presence of a greater number of hydrogen bonds between the turns and bends. Comparatively, the Hvt region of the LH protein presented 5–6 hydrogen bonds, which makes this part of protein more flexible (RMSF values around 3 to 4 Å), with less compaction and stronger fluctuations.

## Discussion

Mealybugs feeding on plants expressing Hvt-lectin fusion proteins showed interesting symptoms. Nymphs were the first to suffer efects of the toxin proteins. This happens because nymphs (1^st^ and 2^nd^ instar) depend mainly on immediate feeding for their growth. Nymphs suck phloem sap to fulfill their dietary needs, while adults have the capability to store some food in their body and are not entirely dependent on feeding during the experimetn. This likely explains the delayed effect of toxin proteins on adult mealybugs compared to nymphs.

Two different proteins Hvt (a spider venom toxin) and onion leaf lectin were fused together to get broad-spectrum resistance against insect pests specifically mealybugs. Both possible fusion constructs were prepared and expressed transiently in a model plant system, tobacco. Both of the single proteins have their own potential applications, but the fusion proteins are more toxic to mealybugs. The plants expressing the fusion protein HL were more effective against mealybugs (85% lethality) than the fusion protein LH (65% lethality), while only 42 and 45% mortalities were recorded on plants expressing parent proteins Hvt and lectin, respectively.

From the proposed models, drastic conformational differences were observed between tertiary structures of both fusion constructs (Fig. 3). Just changing the relative positions of two proteins make a big difference in their structure. The hybrid protein containing Hvt at the N-terminus exhibits more β-motifs rather than α-helices, in contrast to the hybrid protein containing Hvt at the C-terminus, as well as the parent protein. The protein expressing lectin at the N-terminus showed two distinct regions, one has more β-motifs while the other region has almost all the α-helices. This could be an important reason explaining why one protein product is more active against insect pests than the other.

To cope with the issue of sucking pest control, it is important to discover a broad-spectrum solution. In other recent research, different translationally fused proteins were designed for two reasons (i) to control the insect pest and (ii) to enhance the effect of a single protein. The effect of HVIa, Pl1a, and GNA toxicity against *Myzus persicae* (green peach aphid) individually and also as fusion proteins was tested. Fusion proteins proved to be more toxic to aphids than the parent proteins^6^. The Pl1a/GNA fusion protein killed all the individuals of 4106 A strain in 7-days when fed at 1 mg/mL concentration in the diet. For the Hv1a/GNA fusion protein, 100% mortality was observed after 6-days at the same protein concentration^6^. Promising results were collected when fourth instar larvae of *L. oleracea* were fed on the SMI1/GNA fusion protein. Larvae injected with a low concentration of SMI1/GNA (18–25 µg/g insect) were paralyzed and 40% mortality was recorded after 72 h, while 100% mortality was recorded after 48 h at high conc. (60–90 µg/ g insect). In the case of oral intake, 100% mortality rate was found in fifth instar tomato moth larvae when fed on 2.5% fusion protein containing diet^42^. The Butal T/GNA fusion fusion protein proved to be toxic against *Nilaparvata lugens* (rice brown plant hopper). After 6-days of fusion protein uptake 92% mortality was recorded, compared to 64% mortality when feeding on GNA alone^5^.

MD simulations were carried out to predict the core factors responsible for the stability and structural variations found between HL and LH constructs. The RMSD and RMSF plots identified the HL construct as the most stable structure among the tested proteins. The radius of gyration plots confirms the HL protein as a stable and compacted construct with very few fluctuations in its overall structure. In contrast, the LH construct undergoes major conformational changes along the time steps, with greater structural entropy in its structure. These enhanced fluctuations in the LH construct might be attributable to the instability of this protein compared to the other tested proteins.

The calculation of electrostatic interactions of both constructs displays a vague picture regarding the stability of one construct relative to another. However, the detailed visualization and superimposition of post-MD structures of both constructs highlights the Hvt domain as the crucial part responsible for protein stability. Further investigation of the interplay between rigidity and flexibility in terms of stable electrostatic interactions verified the role of the Hvt domain in mediating compactness of the HL construct relative to the LH. The Hvt domain of LH constructs loses its stability because of a smaller number of stable hydrogen bonds among its residues, as compared to the HL construct, which establishes a greater number of electrostatic interactions with more than 50% occupancy through the entire simulation. All of these findings give a glimpse of a strong connection between stability, dynamics and electrostatic forces among a majority of the Hvt domain residues of both fusion constructs.

Based on the above findings, we propose development of transgenic plants with fusion protein having Hvt at the N terminus and evaluation of resistance against multiple insects (aphids, whitefly, jassids etc.) by using either constitutive or phloem-specific promoters.

### Methodology

Two translationally fused proteins were designed to check their effectivity against sucking insect pests. In one construct, Hvt was kept at the N-terminus and lectin at the C-terminus, removing the stop codon of Hvt while in another construct lectin was kept at the N-terminus and Hvt at the C-terminus by removing the stop codon of lectin. This strategy was planned to determine which construct/gene orientation is more effective against sucking pests.

### Cloning of fusion gene constructs

To avoid frame shift of the proteins, both of the gene constructs were synthesized by GenScript using the pUC57 vector backbone. The constructs were then cloned in the expression vector (PVX) using *Sal* I and *Sma* I endonucleases. The individual genes, Hvt and lectin, were also cloned in the PVX vector to compare the effect of the parent toxins with translationally fused toxins.

### Agrobacterium transformation

The final constructs were transformed into *Agrobacterium tumefaciens* strain GV3101 competent cells. Electro-competent cells of GV3101 were prepared and constructs were transformed via electroporation. Colonies were confirmed for the presence of the construct by PCR using gene-specific primers. PCR-positive colonies were picked and cultured in 50 mL LB containing 25 µg kanamycin, 50 µg rifampicin, and 10 µg tetracycline and incubated at 28 °C and 180 rpm for 48 h. This culture was further used for plant infiltration.

### Plant infiltration

After 48 h, cultures were centrifuged in 50 mL polypropylene tubes at 12,000 × g for 10 min. The supernatant was discarded and the pellet was re-suspended in 10 mM MgCl_2_. After re-suspension acetosyringone was added in at a concentration of 100 mM/mL. Plants were infiltrated with the cultures following incubation of 2–3 h at room temperature. *Nicotiana tabacum* was used to carry out all the transient assays. Plants were grown in a plant room at 26 ± 2 °C and 16 L/8 D. plants were infiltrated with a syringe when they reached the 3–4 leaf stage. After infiltration, plants were again kept in the plant room under controlled conditions.

### Insect rearing

Mealybugs were collected from the field and reared in a separate glass house on cotton plants. Mealybugs multiplied readily on cotton plants and were used afterwards for bioassays to check the effects of fusion protein toxins.

### Insect Bioassays

As a result of chlorosis, PVX symptoms appear as mosaic pattern in plants. Usually, it takes 10–14 days for the PVX vector to give visual symptoms in plants. Plants were kept in the cages and mealybugs were allowed to feed at 12 dpi, when plants had clear PVX symptoms. For this purpose, six large cages were used, each carrying 10 plants. The first cage had plants inoculated with the HL construct, the second carried LH-inoculated plants, the third carried Hvt and the fourth carried lectin infiltrated plants. The remaining two cages were used for control plants with which to compare the effects of fusion protein toxins. PVX-inoculated plants were used to see whether the vector impacts the mortality of insects, while healthy *N. tabacum* plants used as experimental controls.

All of the plants in six cages were subjected to feeding by an equal number of mealybugs under controlled conditions of the plant room, *i.e*. 26 ± 2°C and 16 L/8 D. Plants were observed every 24 h to evaluate mealybug performance and collect the mortality data.

### Statistical analysis

ANOVA and Tukey’s SD test were used to assess significance in the mortality data using the statistical software program SPSS-20^43^.

### Computational study of fusion proteins

The sequences of the proteins were subjected to analyses using computational biology tools. This study was carried out primarily to observe conformational/structural modifications produced as a result of the fusion of two proteins.

### Structure validation

The tertiary structures of fusion and parent proteins were predicted by using I-TASSER^38–41^. For further structure validation in detail, 3D coordinates of the best selected models were analyzed. In this regard, RAMPAGE^44^ Ramachandran Plot Assessment was utilized to calculate the Ramachandran plots for each of the four proteins i.e. Hvt, Lectin, HL and LH.

### Molecular dynamics simulation

#### System setup

For the structural refinement and to predict dynamic stability, four validated predicted models, *i.e*. Hvt, lectin, HL and LH proteins, were submitted for extensive MD simulation using AMBER14^45^. All four systems were prepared using xleap module in explicit solvent under periodic boundary condition via application of an FF99SBildn force field. The proteins were solvated in a cubic box of TIP3P water molecules^46^ with a distance of 10 Å between the solute and edge of water box. The perturbed and unperturbed charges of the system were neutralized by adding appropriate number of counter ions.

#### MD simulation

Initially, energy minimization of each system was performed to remove the steric clashes between protein-protein and protein-solvent molecules. A total of 35,000 steps of minimization was carried out involving 15,000 steps each for steepest decent and for conjugate gradient minimization by gradual decreasing the straining force applied on all atoms of protein. Finally, another 5,000 steps of minimization were performed without any restraint to relax the system. It was followed by gradual heating of each system from 0 K to 300 K under NVT conditions for a period of 500 ps. After heating, each system was equilibrated under NPT ensemble (P = 1 atm) for another 500 ps. Finally, a 20 ns production run was conducted for each system at constant temperature and pressure^47^. Particle Mesh Ewald Method (PMEM)^48^ was employed to compute long-range electrostatic interactions with a cut off distance of 8 Å, integration time step of 2 fs and the SHAKE algorithm^49^ was utilized to restrain all bonds involving hydrogen atoms.

The CPPTRAJ module^50^ incorporated in AMBER suite was used for trajectory analysis according to their RMSD, RMSF, radius of gyration, hydrogen bonds and secondary structure plots. During entire simulation period plots presented the comparative stability of fusion constructs over one another.
